# Metformin targets Clusterin to control lipogenesis and inhibit the growth of bladder cancer cells through SREBP-1c/FASN axis

**DOI:** 10.1038/s41392-021-00493-8

**Published:** 2021-03-01

**Authors:** Jun Deng, Mei Peng, Sichun Zhou, Di Xiao, Xin Hu, Simeng Xu, Jingtao Wu, Xiaoping Yang

**Affiliations:** 1 Key Laboratory of Study and Discovery of Small Targeted Molecules of Hunan Province, Department of Pharmacy School of Medicine, Hunan Normal University, Changsha 410013, China; Key Laboratory of Molecular Pharmacology and Drug Evaluation (Yantai University), Ministry of Education, Yantai University, Yantai, 264005 China; 2 Department of Pharmacy, Xiangya Hospital, Central South University, Changsha, 410008 China

**Keywords:** Drug development, Biochemistry, Target identification

**Dear Editor,**

Metformin, a widely prescribed drug for treating type II diabetes, has shown an important anti-cancer property.^1^ However, new launched clinical trials did not achieve such desirable results.^2^ One explanation could be that metformin’s anti-cancer mechanisms and precise therapeutic targets remain unclear. To identify potential therapeutic targets of metformin in bladder cancer, we screened differential proteins with metformin treatment in T24 cells by high-throughput protein chip. As shown in Fig. 1a, Clusterin was screened as a target of metformin in bladder cancer. The direct binding of metformin with Clusterin was further confirmed by Surface Plasmon Resonance (SPR) analysis (Fig. 1b). Meanwhile, results from Western blot and RT-PCR showed that treatment with metformin resulted in significant Clusterin downregulation in UMUC3 and J82 cells (Supplementary Fig. S1a, b). Clusterin is a conserved glycoprotein which plays key roles in cellular stress response and survival.^3^ As evidenced by its key roles in cancer, Clusterin served as a therapeutic target for fighting tumor growth.

**Fig. 1:**
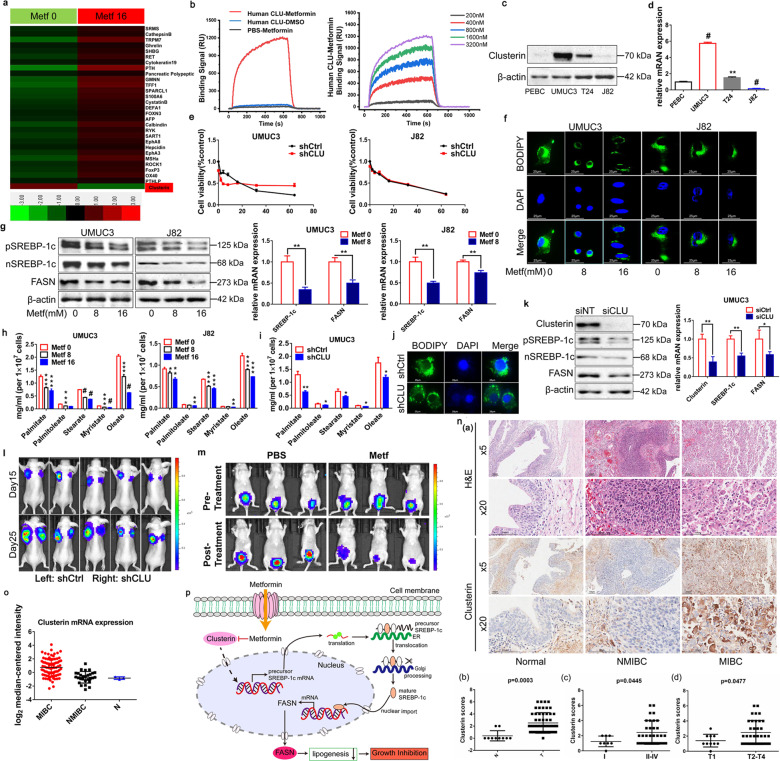
**a** High-throughput protein chip was used to screen differential proteins with metformin treatment in T24 cells for 48 h. Metf 0 metformin 0 mM, Metf 16 metformin 16 mM. **b** SPRi binding signal curve of metformin to Clusterin protein. **c** Western blot analysis of Clusterin protein expression in three human bladder cancer cells and one normal bladder cell (*n* = 3). **d** RT-PCR analysis of Clusterin mRNA expression in three human bladder cancer cells and one normal bladder cell (*n* = 3). **e** MTT was used to assess the cell proliferation after Clusterin knockdown and the effect of metformin on inhibiting proliferation in Clusterin-knockdown cells compared with control cells (*n* = 3). **f** Lipid droplet were visualized using BODIPY 493/503 after metformin treated in UMUC3 and J82 cells (*n* = 3). **g** UMUC3 and J82 cells were treated with metformin for 36 h. Western blot was used to examine the protein expression of SREBP-1c precursor (pSREBP-1c), mature forms of SREBP-1c (nSREBP-1c) and FASN. RT-PCR was used to examine the mRNA levels of SREBP-1c and FASN after treatment with metformin for 6 h (*n* = 3). **h** UMUC3 and J82 cells were treated with metformin for 36 h. GC-MS analyzed the long-chain and short-chain fatty acids levels (*n* = 3). **i** GC-MS determined the long-chain and short-chain fatty acids levels in Clusterin knockdown UMUC3 cells (*n* = 3). **j** Lipid droplet were visualized using BODIPY 493/503 in Clusterin knockdown (shCLU) cells and control cells (shCtrl) (*n* = 3). **k** Protein and mRNA expressions of Clusterin, SREBP-1c, and FASN were detected by Western blot, RT-PCR respectively in UMUC3 cells after siRNA silencing for 36 h. siNT = empty vector; siCLU = Clusterin silence (*n* = 3). **l** The shCtrl and shCLU UMUC3 cells were injected subcutaneously into the left and right flanks of the same nude mice, respectively. Tumor growth was monitored weekly using the IVIS Spectrum imaging system (*n* = 5). **m** Images before and after treatment with metformin or PBS (*n* = 5, each group). Tumor growth was monitored weekly using the IVIS Spectrum imaging system (*n* = 5). **n** Tissue microarray analyzed Clusterin expression in total 66 tissue samples (10 normal tissues and 56 cancer tissues). N normal bladder, NMIBC non-muscular invasive bladder cancer, MIBC muscle-invasive bladder cancer, T bladder tumor. **o** Relative expression of Clusterin mRNA in bladder tumor tissues obtained from TCGA database. **p** A diagram summarizes that metformin inhibits Clusterin-mediated fatty acid synthesis pathway. ER endoplasmic reticulum. *p < 0.05, **p < 0.01, ***p < 0.001, #*p* < 0.0001

We examined the basal level of Clusterin in human bladder cancer cell lines. As shown in Fig. 1c, d, Supplementary Fig. S2a, UMUC3 cells were characterized by significantly highest Clusterin expression while J82 cells were lowest. Interestingly, metformin inhibited cell proliferation, colony formation, migration, and induced apoptosis (Supplementary Fig. S2b–e) greater in higher Clusterin UMUC3 cells compared with lower Clusterin J82 cells.

To determine the direct role of Clusterin in bladder cancer cells growth, we used siRNAs and lentivirus to modulate Clusterin levels in UMUC3 and J82 cells (Supplementary Fig. S3a). Knockdown of Clusterin dramatically suppressed cell viability, migration, colony formation, and induced apoptosis compared with control cells (Fig. 1e, Supplementary Fig. S3b–d). We next examined the effect of metformin with Clusterin-silencing conditions. Figure 1e showed that in Clusterin knockdown UMUC3 cells, low concentration of metformin (0–8 mM) inhibited proliferation stronger than in parental cells, indicating that both knocking down Clusterin and metformin treatment inhibited cellular proliferation in a synergetic fashion. However, when the concentrations of metformin (8–64 mM) were high, metformin itself reduced the expression of Clusterin, making the loss of Clusterin in these cells almost completely. Thus, the inhibitory effect of metformin in Clusterin knockdown cells is inversely lower than in parental cells, suggesting that anticancer effect of metfornin relies on Clusterin expression. Therefore, in lower Clusterin J82 cells, knocking down of Clusterin did not induce any significant decrease in the inhibitory effect of metformin (Fig. 1e). Metformin also significantly inhibited basal migration, colony formation, and induced apoptosis in control cells. However, these effects of metformin in Clusterin knockdown cells were significantly reduced (Supplementary Fig. S3b–d). We also successfully induced Clusterin overexpression in UMUC3 and J82 cells (Supplementary Fig. S3e). Bladder cancer cells with stable Clusterin overexpression led to increased cellular proliferation, colony formation and migration (Supplementary Fig. S3f–h). Meanwhile, cells with stable Clusterin overexpression were characterized by higher metformin induced inhibition of proliferation, colony formation as well as migration compared with control cells (Supplementary Fig. S3f–h). Taken together, these results suggested that bladder cancer cells characterized by higher Clusterin are more sensitive to metformin induced inhibition of growth.

Previously, we found that metformin reduced FASN expression in bladder cancer cells.^4^ Furthermore, FASN and SREBP-1c were significantly higher in bladder cancer cell lines and tissues (Supplementary Fig. S4a, b). Therefore, we systematically investigated the function of metformin in bladder cancer lipogenesis. Lipid-droplet staining showed that metformin, dramatically decreased the number, size, and staining intensity of lipid bodies both in UMUC3 and J82 cells (Fig. 1f, Supplementary Fig. S4c). Meanwhile, SREBP-1c and its downstream effectors were significant decreased (Fig. 1g, Supplementary Fig. S4d). We also conducted lipid metabolite analysis with metformin treatment via Gas Chromatography-Mass Spectrometer (GC-MS). As shown in (Fig. 1h), the contents of long-chain and short-chain fatty acids were significantly decreased in comparison to that of the controls. Since Clusterin participates in lipid transport and metabolism,^5^ we speculated that whether Clusterin plays roles in metformin caused lipogenesis inhibition. As shown in Fig. 1i, the depleted levels of fatty acids correlated with the striking reduction in Clusterin expression. Meanwhile, knocking down Clusterin decreased cellular neutral lipid levels and both protein and mRNA expressions of SREBP-1c and its downstream effectors (Fig. 1j, k, Supplementary Fig. S4e, f), suggesting that silencing Clusterin restrained the transcriptional activity of SREBP-1c and inhibited lipogenesis. Furthermore, double immunofluorescence staining showed Clusterin was distributed in the cytoplasm while SREBP-1c located in both cytoplasm and nucleus (Supplementary Fig. S4g). Knockdown Clusterin significantly reduced SREBP-1c in both nucleus and cytosol (Supplementary Fig. S4h). However, CO-IP showed no binding between Clusterin and SREBP-1c in cells (Supplementary Fig. S4i). Taken together, these results confirmed that metformin reduced formation of neutral lipid which is mediated by Clusterin.

To investigate the role of Clusterin in tumorigenesis, subcutaneous tumors bearing stable knockdown Clusterin (UMUC3-shCLU) and parental UMUC3 cells (UMUC3-shCtrl) were implanted, respectively. The tumors derived from UMUC3-shCLU cells resulted in a remarkably slower growth rate (Supplementary Fig. S5a), smaller tumor size, and weight (Fig. 1l, Supplementary Fig. S5b). Finally, we confirmed that Clusterin, FASN, and SREBP-1c protein levels were decreased in UMUC3-shCLU cell developed tumor tissue (Supplementary Fig. S5c). These results suggest that Clusterin depletion can effectively suppress bladder tumor xenografts in vivo.

Given the potent inhibitory effects of metformin in bladder cancer cell growth, we evaluated its antitumor activity in vivo using nude mice bearing UMUC3-luc orthotopic models (Supplementary Fig. S6a). Intravesical metformin substantially decelerated tumor growth (Fig. 1m) and significantly reduced the weight of bladders (Supplementary Fig. S6b, c). Furthermore, metformin treated mice showed reduced lipogenesis (Supplementary Fig. S6d). H&E and Ki67 staining demonstrated that metformin effectively inhibited tumor growth (Supplementary Fig. S6d). Meanwhile, we found that metformin decreased Clusterin, SREBP-1c, and FASN levels (Supplementary Fig. S6d). In summary, we demonstrated that metformin blocked bladder cancer growth and inhibited lipogenesis-associated proteins in orthotopic models which were established by high expressed Clusterin UMUC3 cell line.

To explore the correlation between Clusterin and bladder cancer progression, we first examined its expressions in bladder tumor patient specimens. Immunohistochemistry analysis of tissue microarray showed that Clusterin protein level was significantly higher in bladder cancer compared with normal bladder (*p* = 0.0003) (Fig. 1n (a, b)). In particular, it expressed higher in muscle invasive bladder cancer compared with non-muscle invasive bladder cancer (*p* < 0.05) (Fig. 1n (a)), consistent with the results of TCGA database and clinical samples (Fig. 1o, and Supplementary Fig. S7). Immunohistochemistry analyses showed that Clusterin level was positively correlated with tumor stage and differentiation grade (Fig. 1n (c, d)). These results implied that Clusterin expression positively correlates with bladder cancer progression and may serve as a biomarker for treating with metformin in a personalized medicine fashion.

In summary, our study proved that Clusterin is a target of metformin and the susceptibility to Clusterin-depended growth inhibitory effects of metformin in bladder cancer. Moreover, metformin targets Clusterin and inactivates SREBP-1c and its downstream target FASN, leading to blockage of de novo fatty acid synthesis to inhibit growth of bladder cancer (Fig. 1p). These results uncovered an important role for Clusterin-regulated lipogenesis in maintaining bladder tumor growth and survival. This study provides a novel potential approach for individualized treatment with metformin in cancer patients with differential expression of Clusterin.

## Supplementary information

Clusterin-Supplementary materials

## Data Availability

The data sets used and/or analyzed during the current study are available from the corresponding author on reasonable request.
