# Supplementary material for: Metformin targets Clusterin to control lipogenesis and inhibit the growth of bladder cancer cells through SREBP-1c/FASN axis
Source: Signal Transduct Target Ther. 2021 Mar 1;6:98. doi: 10.1038/s41392-021-00493-8 (PMC7921554; doi:10.1038/s41392-021-00493-8)
Supplement: Supplementary file 1 — Clusterin-Supplementary materials [file 41392_2021_493_MOESM1_ESM.docx]

Supplementary Materials for

Metformin targets Clusterin to control lipogenesis and inhibit the growth of bladder cancer cells through SREBP-1c/FASN axis

Jun Deng^1#^, Mei Peng^1,2#*^, Sichun Zhou^1^, Di Xiao^1^, Xin Hu^1^, Simeng Xu^1^, Jingtao Wu^1^, and Xiaoping Yang^1*^.

Correspondence to: Xiaoping Yang (Xiaoping.Yang@hunnu.edu.cn) or Mei Peng (Meipeng@csu.edu.cn)

**This PDF file includes:**

Materials and Methods

Figures. S1 to S7

Materials and Methods

**Reagents**

Metformin was purchased from Aladdin chemistry Co. Ltd (Houston, TX, USA). It was prepared in a range of concentrations in culture medium.

Antibodies were purchased from the following suppliers: anti-FASN, anti-β-actin (Cell Signaling Technology, Beverly, MA, USA); anti-Clusterin, anti-SREBP-1c (Santa Cruz Biotechnology, Dallas, TX); anti-Histone-H3, anti-Alpha Tubulin (Proteintech, USA).

**Cell lines and culture conditions**

The human bladder cancer cell lines UMUC3, J82 and T24 were purchased from iCell Bioscience Inc (Shanghai, China) and the normal human bladder cells PEBC provided by Dr. P Guo. All cell lines were cultured in MEM (Hyclone, Logan, UT, USA) supplemented with 10% fetal bovine serum (FBS; Hyclone) and 1% penicillin–streptomycin. Cultures were incubated at 37℃ in humidified air containing 5% CO_2_.

**Human** **urothelial carcinoma tissues**

Tissue microarray consisting of 10 normal tissues and 56 cancer tissues (National Human Genetic Resources Sharing Service Platform, 2005DKA21300） were analyzed by Shanghai Outdo Biotech Co., Ltd. Urothelial carcinoma tissues samples were used as previously described^1^. Consents from all patients were obtained. Approval of this study was obtained from the Research Ethics Board at Xiangya Hospital (No. 201703229).

**Protein Array analysis**

The T24 cells were treated with metformin for 48 h subjected to RayBiotech antibody array with 493 human proteins for the candidate biomarker screening, data collection and analyses were conducted by RayBiotech, Guangzhou, China. This chip is based on an antibody array, and protein abundances were measured.

**SPRi interaction and affinity analysis**

To understand the specific interactions between metformin and Clusterin protein, we performed an affinity measurement using surface plasmon resonance (SPRi) technology. The SPRi validation experiment was performed with the bScreen LB 991 Label-free Microarray System (Berthold Technologies, Germany). Data collection and analyses were conducted by BetterWays, Guangzhou, China.

**Cell viability assay and cologenic assay**

Cell viability was assessed using a tetrazolium-based assay. Briefly, cells were seeded at 8 × 10^3^ per well in 96-well culture plates and incubated in medium containing 10% FBS. After 24 h cells were treated with different concentrations of metformin for 48 h. The tetrazolium salt of MTT (20 μl; Sigma) was dissolved in PBS to a concentration of 5 mg/ml, and then added to each well. The plates were incubated another 5 h. The medium was aspirated from each well, 150 μl DMSO was added to dissolve formazan crystals, and absorbance was measured using a microplate reader (Biotek, SYNERGY HTX, VT, USA) at 490 nm. Dose-response curves were generated and used to calculate the half-maximal inhibitory concentration (IC_50_) using SPSS 16.0 (IBM, Chicago, IL, USA).

Cologenic survival was defined as the ability of the cells to form colonies. Brieﬂy, 8 × 10^3^ cells were seeded into 24-well dishes in 0.5 ml of medium. At 24 h, cells were treated with metformin for a further 7-day period in 1ml medium containing 10% FBS. Cells were fixed with 0.5 ml 10% formaldehyde and stained with 0.1% crystal violet. Absorbance was measured using a microplate reader (Biotek, SYNERGY HTX) at 550 nm.

**Immunoprecipitation**

Cell lysis was carried out using lysis buffer (Beyotime, China). After centrifugation, the supernatant was collected. The antibodies were added to lysates with protein A/G beads (Santa Cruz). Samples were incubated overnight. The beads were collected by centrifugation, the beads were then washed three times using IP buffer. Sample loading buffer (5×) was mixed with the beads and boiled for 10 min. The supernatant was used for western blot analysis.

**RNA extraction and quantitative real-time PCR**

Total RNA was isolated using a RNeasy mini kit (QIAGEN, Beijing, China). Complementary DNA (cDNA) was synthesized using a high-capacity cDNA reverse transcription kit (Thermo, Shanghai, China). Quantitative real-time polymerase chain reaction (qRT-PCR) was carried out using a TaqMan Gene Expression Master Mix (Bio-Rad, Shanghai, China) according to manufacture protocol and TaqMan probes for human Clusterin (Sangon Biotech, Shanghai, China), and human glyceraldehyde-3-phosphate dehydrogenase (GAPDH) (Sangon Biotech, Shanghai, China). The custom-made primers for RT-PCR analysis of other genes were as follows: Clusterin (FP-ATTCATACGAGAAGGCGACG, RP-CAGCGACCTGGAGGGATT); FASN (FP-TGCCCTGAGCT GGACT ACTT, RP-AAAGCTGCTCAGGACCATGT); SREBP-1c (FP-GACAG CCCAGTCTTTGAGGA, RP-CAGGACAGGCAGAGGAAGAC); GAPDH (FP-CAAGGTCATCCATGACAACTTTG, RP-GTCCACCA CCCTGTTGCTGTAG). ACC (FP-TACCTTCTTCTACTGGCGGCTGAG, RP-GCCTTCACTGTTCCTTCCACTTCC); SCD-1 (FP-GCGATATGCTGTGGTGCTTA, RP-AGCTCCAAGTGAAACCAGGA).

**siRNA transfection**

UMUC3 and J82 cells were transfected with commercially available Clusterin siRNA (siCLU-1, 5’-GCAGCAGAGUCUUCAUCAU-3’; siCLU-2, 5’-CAGCAGCAGAGTCTTCATCAT-3’; Ribobio, China) or negative control siRNA (siNT, 5’-UUCUCCGAACGUGUCACGUTT-3’; Ribobio, China) with the transfection reagent Lipofectamine 2000 (Invitrogen). Briefly, cells were plated in six-well plates at a density of 3 × 10^5^ cells per dish, cells at 30%-50% confluence first transfected with 50 nM Clusterin siRNA or 50 nM negative control siRNA using Lipofectamine 2000 in the absence of antibiotics or FBS for 6 h. Following a washing in PBS, the medium was replaced with MEM for an additional 36 h. Cell protein was collected and specific silencing was confirmed by western blot analysis.

**Knockdown or overexpression of Clusterin by transfection**

The Clusterin knockdown lentiviral vectors shCLU-1 (69466-1), shCLU-2 (69467-1), CON206, and Clusterin overexpression lentiviral vectors LV-CLU (7356-2), CON254 were constructed by GeneChem Co. Ltd. (Shanghai, China). Brieﬂy, Prior to the transfection, the cells (2 × 10^5^) were plated in six-well plates and cultured for 24 h until they grew to 30-40% confluency. Subsequently, the lentiviruses were added to the wells with 1 ml of MEM and 40 μl Transfection reagent (GeneChem, Shanghai, China). After incubation for 12 h, the medium containing the virus was removed and replaced with normal MEM. Then, knockdown or overexpression efficiency was verified by western blot analyses.

**Apoptosis**

Apoptosis Detection kits (FITC Annexin V) were purchased from BD Pharmingen, apoptosis was assessed using flow cytometry in a separate experiment. Briefly, cells were treated with different concentrations of metformin for 24 h, then harvested with trypsinization, washed twice with phosphate-buffered saline (PBS), and resuspended in 1× binding buffer to 1 × 10^6^ cells/ml. Transfer 100 µl of the solution (1 x 10^5^ cells) to a 5 ml culture tube. Add 5 µl of FITC Annexin V and 5 µl PI. Gently vortex the cells and incubate for 15 min at RT (25℃) in the dark. Add 400 µl of 1× Binding Buffer to each tube. Analyze by flow cytometry within 1 h, labeled cells were counted by flow cytometry on a FACS Calibur flow cytometer [excitation wavelength, 488 nm; emission wavelengths, 530 nm (FL-1 channel, FITC) and 670 nm (FL-3 c3 channel, propidium iodide)]. Data were analyzed using Cell Quest software (Becton–Dickinson). Non-apoptotic cells were defined as those negative for Annexin-V and propidium iodide; necrotic/late apoptotic cells as those positive for both labels; and early apoptotic cells as those positive for Annexin V but negative for propidium iodide.

**Western blotting**

Tissues and cell proteins were fractionated by SDS-PAGE, transferred to membranes, and then incubated overnight at 4℃ with different primary antibodies described in Reagents section above (Cell Signaling, Beverly, MA, USA) in buffer containing bovine serum albumin (BSA). Membranes were washed with TBS containing 0.05% Tween-20, blotted with secondary antibody for 1 h at room temperature, then washed again three times. Pierce Super Signal chemiluminescent substrate (Rockford, IL, USA) was added, and the blot was imaged immediately on a ChemiDoc system (Bio-Rad, Hercules, CA, USA) and a Perfection V500 camera (Epson). Band intensities were quantified using Image J.

**Immunofluorescence staining**

UMUC3 cells were grown on a coverslip to 80% confluence. Cells were fixed by 4% paraformaldehyde for 10 min. 0.1% Triton X-100 was used to increase antigen accessibility. Cells were blocked with 1% bovine serum albumin for 1 h and incubated with primary antibodies overnight at 4℃. Cells were washed using PBS and incubated with DyLight 549 (Proteintech, USA) or Alexfluor 488 (Proteintech, USA) labeled secondary antibody for 60 min at room temperature, and stained with DAPI (4’,6-diamidino-2-phenylindole solution, Proteintech, USA) for 5 min. For lipid-droplet staining, fixed UMUC3 cells were incubated with 1 μg/ml BODIPY™493/503 for 30 min (Invitrogen, Eugene, USA). Cells were washed using PBS, mounted with Prolong Gold antifade reagent containing DAPI (Invitrogen, Carlsbad, CA). Immunofluorescence images were captured using an OlympusFV3000 confocal microscope.

**Transwell invasion assay**

Cell migration ability was assessed by transwell plates (Corning). About 4 × 10^4^ cells in 200 µl serum-free medium were seeded into the top chambers, and 700 µl medium supplemented with 20% serum was used as a chemo attractant in the lower chamber. After incubating for 24 h at 37℃ in 5% CO_2_, the medium in top chambers was removed, and the cells were fixed with 4% paraformaldehyde and stained with 0.1% crystal violet. The non-migrating cells on the upper sides of membrane were removed by cotton wool. The cells on the lower sides of membrane were counted. Five random fields (×200) were selected and calculated the average. The data represent the mean ± standard error of three independent experiments.

**FA composition assays**

The fatty acid composition was analyzed using Agilent 7890A/5975C equipped with VF-23 ms (Agilent) column: 30 m, (length), I.D. 0.25 mm wide bore, film thickness of 0.25 μM as previously described^2^. Briefly, 750 μl mixture of dichloromethane and methanol (2:1) and 100 μM butylated hydroxytoluene (to prevent lipid peroxidation) were added to the cell sample, and homogenized. The mixture was then added with 250 μl dichloromethane and blended for 30 s, and then 250 μl water was added and blending was continued for another 30 s. We transferred the lower phase and subsequently evaporated to dryness by nitrogen. The residue was dissolved by *n*-hexane, and then 2 ml of 0.5 M KOH-MeOH was added, the sample was heated at 60 ℃ for 20 min. Following 10 min of cooling period, 3 ml of 12.5% H_2_SO_4_ in methanol was added to methylate the sample. After an additional 60 min of heating in the water bath (60 ℃), the sample vial was left at room temperature to cool down, and 1 ml of saturated solution containing sodium chloride and 2 ml of hexane was added. After lipid extraction and purification, the hexane fraction was then transferred for GC analysis. Fatty acid identification was identified by comparison of their retention times with authenticated fatty acid methyl ester standards (Supelco 37, Sigma). Fatty acid composition data was expressed as percentage of peak area and each sample was analyzed in triplicates.

**Cytosolic/nuclear fractionation**

Detected cells cytoplasm and nucleus protein expressions by the Nuclear and Cytoplasmic Extraction Kit (CWBIOTECH, Beijing, China), according to the manufacturer’s protocol. Cells 1 × 10^7^ were lysed in 1ml Nc-Buffer A incubate on the ice for 20 min. Then add 55 μl Nc-Buffer B incubate on the ice A for 1 min. Samples were centrifuged at 1,2000 rpm for 15 min at 4℃. The cytosolic supernatant fraction was collected on ice. The nuclear pellet was resuspended in Nc-Buffer C incubate on the ice for 40 min and centrifuged at 1,2000 rpm for 15 min at 4℃. Supernatant fractions were collected and analyzed, or stored at -80℃ for further use.

**Preparation of UMUC3-luc cells**

For imaging purposes, the firefly luciferase gene was transfected into UMUC3 cell line using lentiviral construct as previously^3^ described. Briefly, the full-length luciferase-coding DNA sequence was amplified by PCR reaction from the pGL-3 vector (Promega) and inserted at BamHI and NotI sites of the lentiviral vector pLEX. In addition, Kozak sequence GCCACC was added between BamHI and start codon ATG of luciferase to ensure the luciferase expressed correctly. To generate luciferase-expressing lentivirus (Lentiluc), this vector was co-transfected using calcium phosphate precipitation method with packaging plasmid psPAX2 and envelope plasmid pMD2.G into HEK-293T cells. Supernatant was collected and filtered with 0.45-μm microfilter for infection. UMUC3 cells were then infected with Lenti-luc virus (25 μl viral supernatant/ml medium) and mixed with polybrene (4 μg/ml medium). A single clone with strong luminescent intensity was selected with puromycin and subcultured. Approximately passages 3 to 4 UMUC3-luc cells were used for further work.

**Xenograft tumor mouse model**

Female BALB/c nude mice were purchased from Hunan SJA Laboratory Animal Co., Ltd (Changsha, Hunan, China). All our animal experiments were conducted in accordance with guidelines approved by the Institutional Animal Care and Use Committee at Hunan Normal University (Protocol 2019047). Female BALB/c nude mice (6 weeks old, 18.0 ± 2.0g) were subcutaneously injected with the single-cell UMUC3 lines carrying inducible lentiviral shRNA vector targeting Clusterin (right flank, shClu) or with inducible lentiviral control shRNA vector (left flank, shCtrl) (1 × 10^6^ cells in 100 μl PBS, day 1). Tumor size was measured using caliper, and tumor volume was determined by using the formula: 1/2 × [length × (width)^2^]. Tumor growth was measured using the IVIS Spectrum imaging system. After 25 days, all mice were euthanized.

Orthotopic Implantation: Exponential growth of UMUC3-luc cells (transfected with luciferase) was harvested, and cell density in collection tube was counted by cell counter. Female BALB/c nude mice 6 to 8 weeks of age were used for cancer cell implantation. Brieﬂy, 3 × 10^6^ UMUC3-luc cells in 50 µl PBS were injected into the bladder wall using 1ml syringes and catheter scratching according to the previously described protocol^3^. Female BALB/c nude mice with orthotopic bladder cancer were randomly divided into two groups, which received an instillation of 50 µl PBS, or 50 µl 320 mM metformin intravesically (n = 5, each group). All treatments started at day 2 post-tumor implantation, twice per week, and continued for 2 weeks. Tumor burden was checked weekly through Xenogen IVIS (In Vivo Imaging System) (PerkinElmer, Waltham, MA, USA). Mice were injected with Luciferin approximately 7 min prior to imaging.

**Oil Red O Staining**

Tissue sections were fastened with 4% paraformaldehyde (Servicebio, Wuhan, China) for 20-30 min and then washed with PBS. After washing, tissue sections were cultured with 60% isopropanol for 30 s and then Oil Red O (Solarbio, G1262, China) incubated for 15 min at room temperature. The staining solution of tissue sections was cleaned, then stained with hematoxylin stain (Servicebio, Wuhan, China) for 5 min.

**Histology**

Bladders were fixed in 4% paraformaldehyde (Servicebio, Wuhan, China) 24 h, wax embedded in paraffin and sectioned to a thickness of 7 μm. For histological analysis, the sections were washed three times with distilled water for 3 min. Sections were stained with haematoxylin and eosin (H&E) (Servicebio, Wuhan, China) and assessed for disease grading. For immunohistochemistry staining, sections were deparaffinised and rehydrated using xylene, 100%, 95% then 75% ethanol and Sections were incubated with 3% H_2_O_2_ for 20 min to block endogenous peroxidase activity, washed with PBS and boiled in Tris-EDTA retrieval solution for 5 min in a pressure cooker. Cool naturally to room temperature. Sections were incubated overnight at 4℃ with primary antibody anti-Ki-67, anti-Clusterin, anti-SREBP-1c anti-FASN. Sections were washed with PBS and incubated with Reagent 2 (Goat hypersensitivity two-step detection kit (ZSGB-BIO, Beijing, China) for 20min at room temperature. Sections were then washed with PBS and incubated for 20 min with Reagent 3 (Goat hypersensitivity two-step detection kit (ZSGB-BIO, China, Beijing)) according to manufacturers’ instructions. Sections were washed with PBS and stained using the DAB substrate Kit from Cell Signaling Technology (Cell Signaling, Beverly, MA, USA), counterstaining with Gill’s haematoxylin (Solarbio). Sections were then dehydrated and mounted using neutral resins (Solarbio).

**Statistical Analyses**

All data are presented as mean ± SD. Statistical analysis was performed using SPSS 16.0 (IBM, New York, USA). Differences between groups were assessed for significance using Student t test for experiments involving only two groups and using analysis of variance and the least significant difference test for experiments involving more than two groups. Graphs were generated using GraphPad Prism 6.0. Differences of **P* < 0.05 were considered statistically significant.

**References**

1 Su, Q. et al. Down-regulation of PKM2 enhances anticancer efficiency of THP on bladder cancer. Journal of Cellular and Molecular Medicine 22, 2774-2790 (2018).

2 Tang, M. et al. The status of omega-3 PUFAs influence chronic unpredicted mild stress-induced metabolic side effects in rats through INSIG/SREBP pathway. Food & Function 10, 4649-4660 (2019).

3 Yang, X. et al. Diphtheria Toxin-Epidermal Growth Factor Fusion Protein DAB(389)EGF for the Treatment of Bladder Cancer. Clinical Cancer Research 19, 148-157 (2013).

Figure. S1.


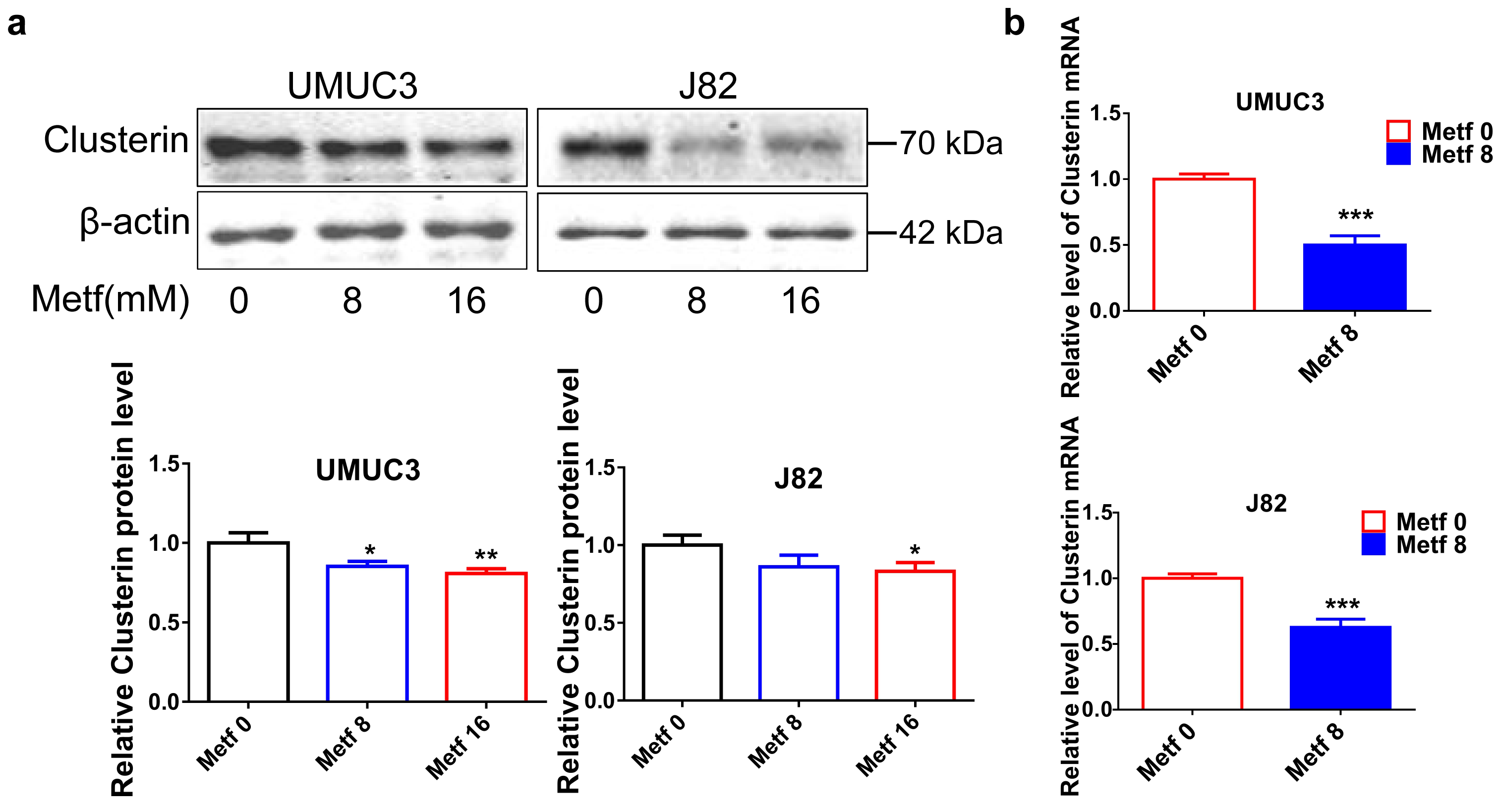


Figure. S1 Metformin treatment decreases Clusterin expression in bladder cancer cells. a Western blot analysis of Clusterin protein expression after treatment with metformin for 36 h. b RT-PCR analysis of Clusterin mRNA expression after treatment with metformin for 6 h. Metf 0=metformin 0 mM; Metf 8=metformin 8 mmol. **p* < 0.05, ***p* < 0.01, ****p* < 0.001 (n=3).

Figure. S2.


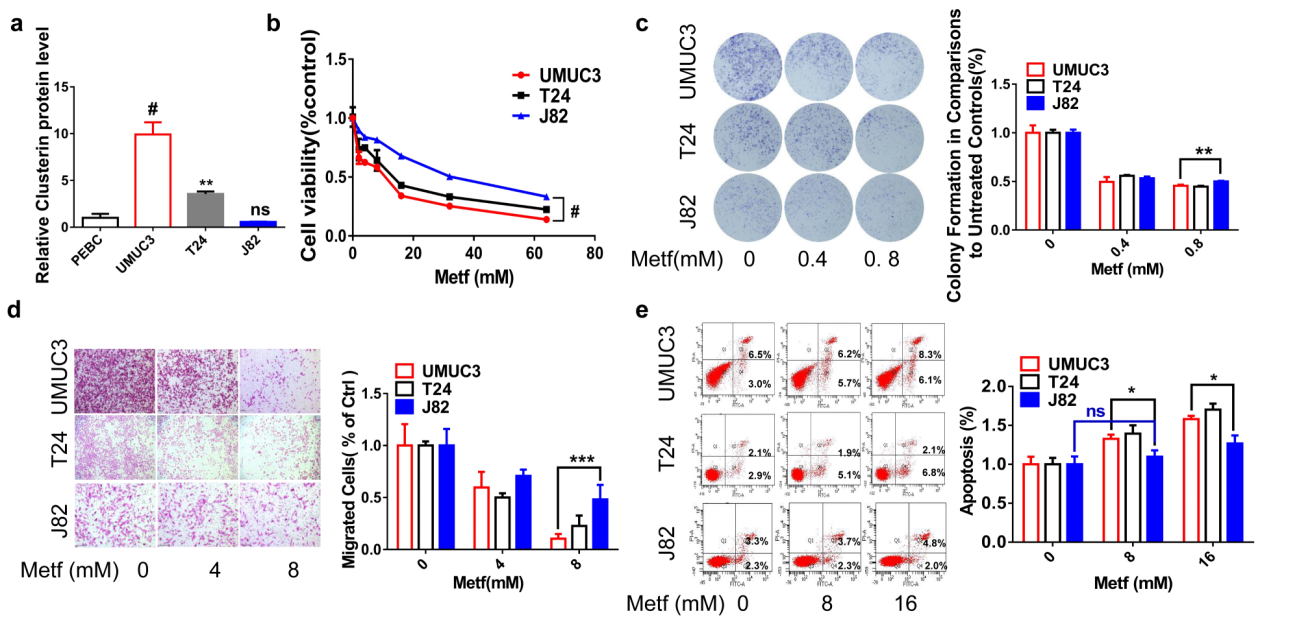


**Figure. S2 Clusterin was differentially overexpressed in bladder cancer cell lines and higher expressed Clusterin cells were more sensitive to metformin treatment.** **a**. Relative levels of Clusterin protein expression in three human bladder cancer cells and one normal bladder cell (n=3). **b**. Effect of metformin on bladder cancer cell proliferation. Viability of UMUC3, T24 and J82 cells was assessed by MTT. **c**. Evaluation of colony suppression by metformin on three bladder cancer cell lines. **d**. Transwell assay was used to assess migration after metformin treatment. **e.** Flow cytometry detected cell apoptosis after metformin treatment. Metf stands for metformin. **p* < 0.05, ***p* < 0.01, ****p* < 0.001, # *p* < 0.0001, ns, not significant (n=3).

Figure. S3.


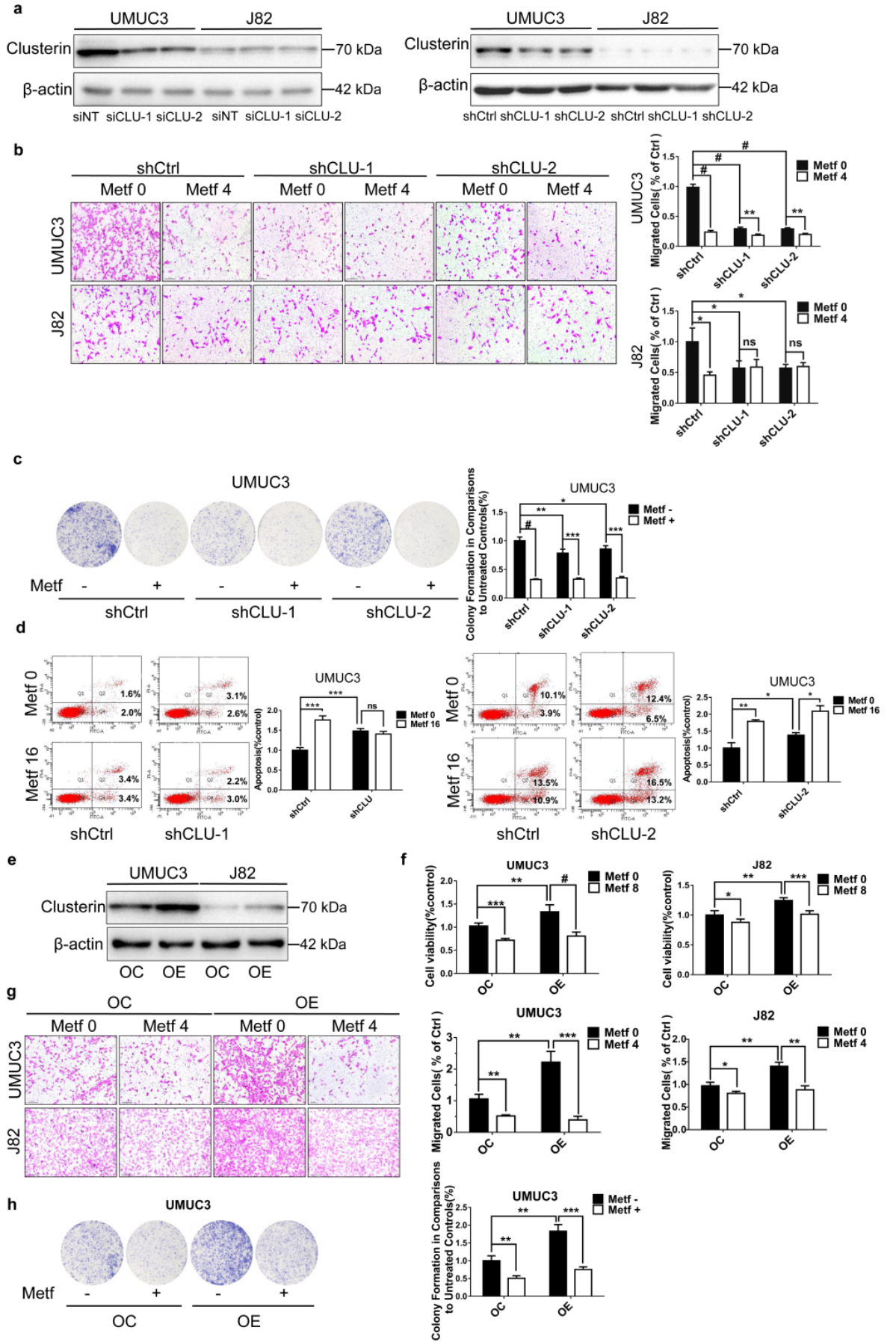


Figure. S3 Inhibitory effect of metformin is associated with Clusterin expression levels in bladder cancer cells. a The expression of Clusterin was measured by western blot after transfection with negative control siRNA (siNT) or siClusterin (siCLU-1, siCLU-2) and nonsense shRNA (shCtrl) or two different shRNAs (shCLU-1 and shCLU-2) targeted against human Clusterin gene. b, c, d. transwell assays, colony formation assay and flow cytometry were used to assess the cell migration, colony suppression and apoptosis after Clusterin knockdown and the effect of metformin on inhibiting migration, colony formation and apoptosis in Clusterin-knockdown cells compared with control cells. Metf 0=metformin 0 mM; Metf 4=metformin 4 mM; Metf 16=metformin 16 mM; Metf - = metformin 0 mM; Metf + = metformin 0.4 mM. e. Overexpression of Clusterin in UMUC3 and J82 cells was measured by western blot. OC=control cells transfected with empty vector; OE=cells with Clusterin-overexpression. f, g, h. MTT, transwell assays and colony formation assay were applied to assessed the cell proliferation, migration and colony suppression after Clusterin overexpression and the effect of metformin on inhibiting proliferation migration and colony formation in Clusterin overexpression cells compared with control cells. **p* < 0.05, ***p* < 0.01, ****p* < 0.001, # *p* < 0.0001, ns, not significant (n=3).

**Figure. S4.**

**
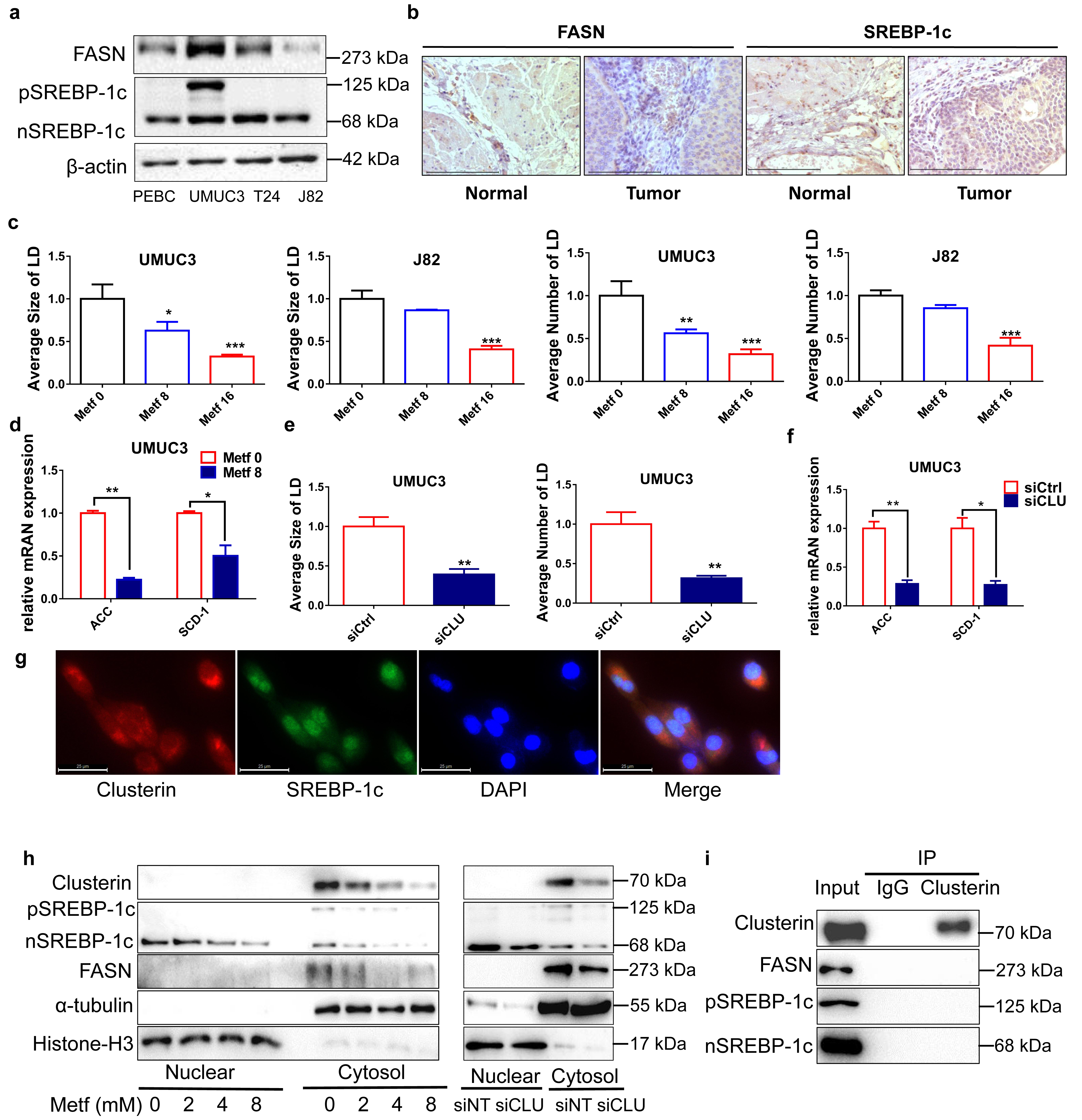
**

**Figure. S4 Metformin downregulated lipogenesis via SREBP-1c and FASN axis. a,** Western blot analysis of FASN and SREBP-1c protein expression in three human bladder cancer cells and one normal bladder cell. **b** Immunohistochemistry was used to detect the expression of SREBP-1c and FASN in human bladder cancer tissues and adjacent normal tissues (n=5). **c** Statistics analysis of lipid droplets size and number in UMUC3 or J82 treated with metformin. LD = lipid droplets. **d** RT-PCR analysis was used to examine the expression of ACC and SCD-1 mRNA after treatment with metformin for 6 h. **e** Statistics analysis of lipid droplets size and number in UMUC3 cells after siRNA silencing for 36 h. **f** RT-PCR analysis was used to examine the expression of ACC and SCD-1 mRNA in UMUC3 cells after siRNA silencing for 36 h. **g** Clusterin and SREBP-1c localization were detected by immunofluorescence co-staining in UMUC3 cells. **h** UMUC3 cells were treated with metformin for 36 h or Clusterin siRNA, cell lysates were collected and separated into nuclear and cytoplasmic fractions, and the protein levels of Clusterin, pSREBP-1c, nSREBP-1c and FASN were detected by western blot. **i** Coimmunoprecipitation (IP) assays was used to detect the interaction between Clusterin and SREBP-1c in UMUC3 cells with anti-Clusterin and anti-SREBP-1c antibodies, respectively. Immunoprecipitation using normal IgG served as controls. **p* < 0.05, ***p* < 0.01, ****p* < 0.001 (n=3).

**Figure. S5**

**
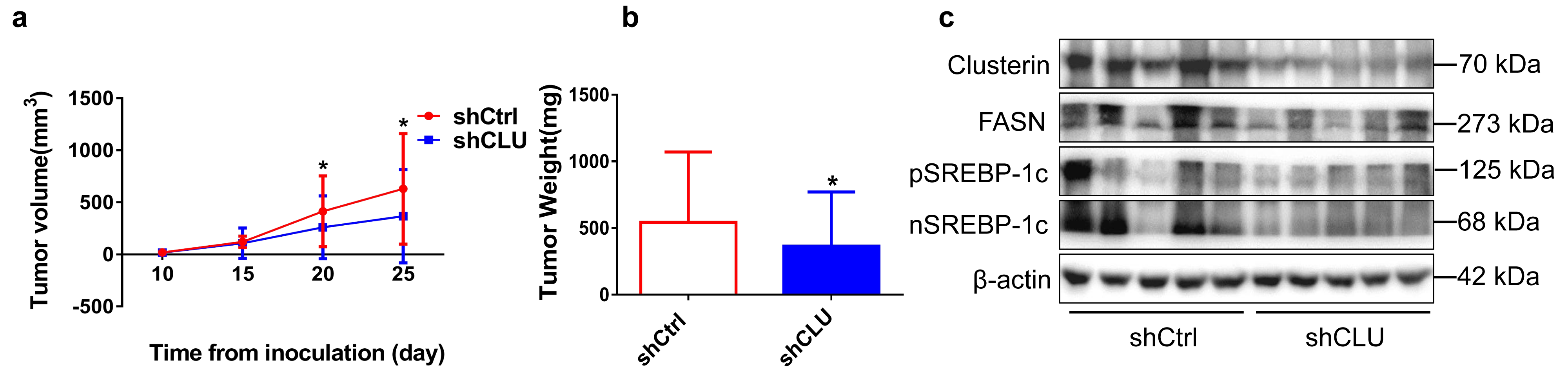
**

**Figure. S5 Clusterin depletion reduced the growth of bladder cancer cells in nude xenografts mice.** **a** The tumor volume of shCtrl and shCLU groups of mice was measured at indicated time. **b**. The tumor weights of shCtrl and shCLU groups of mice were measured. **p*< 0.05 (n=5). **c**. The expressions of Clusterin, FASN and SREBP-1c were detected by western blot in UMUC3-shCtrl cell and UMUC3-shCLU cell developed tumor tissue.

**Figure. S6**

**
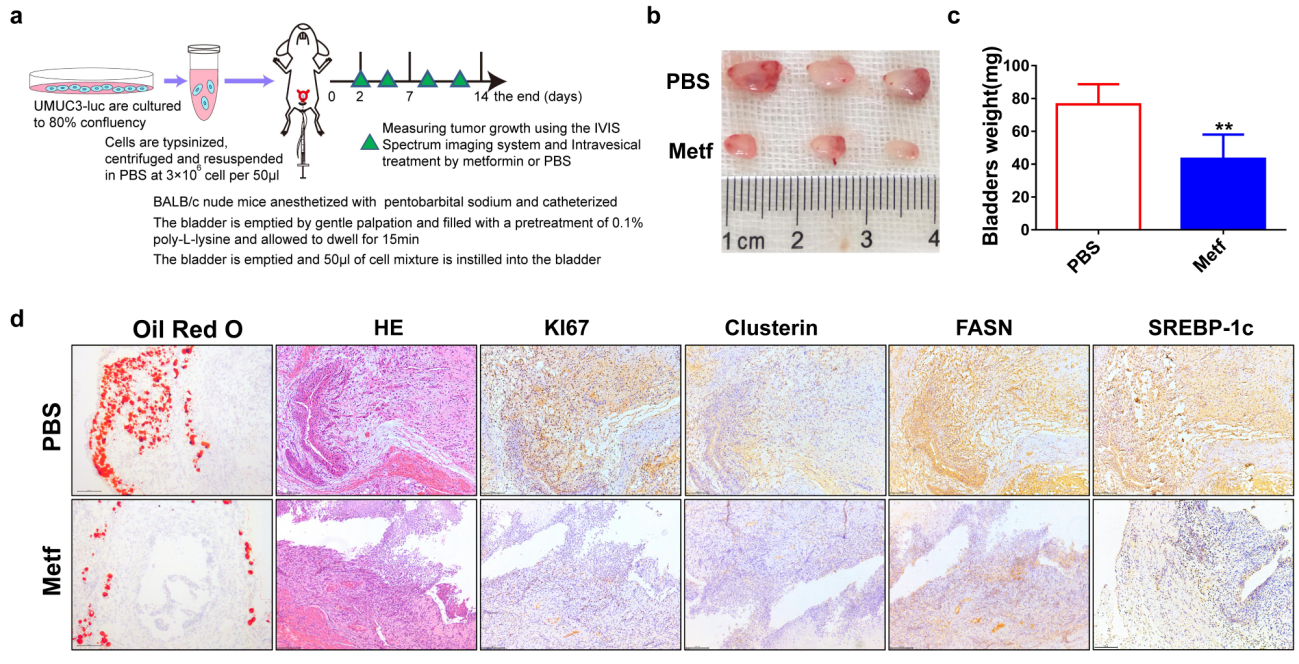
**

**Figure. S6 Intravesical metformin profoundly suppressed tumor growth in orthotopic models in highly Clusterin expressed bladder cancer cells.** **a** Schematic of the protocol used to instill UMUC3-luc cells. **b** The images of the explanted bladder tumors. **c**. Weights of mouse bladders including those that died before the end of experiment were measured. Shown are means and SD. ***p* < 0.01 (n=5). **d**. Bladder tumor tissues were subjected to oil red O, H&E, ki67 staining and immunostaining for Clusterin, FASN and SREBP-1c.

**Figure. S7**

**
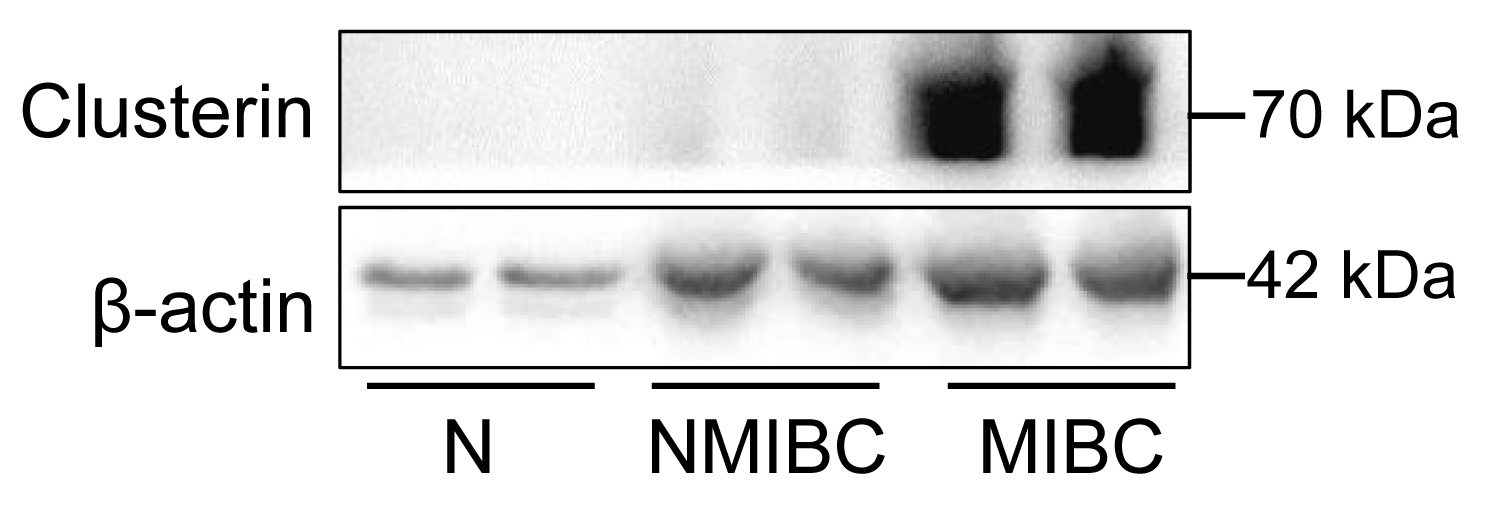
**

**Figure. S7.** The representative protein expression of Clusterin in human bladder cancer tissues and adjacent normal tissues (n=5). N=normal bladder; NMIBC=non-muscular invasive bladder cancer; MIBC=muscle-invasive bladder cancer.
